# Absence of toll-like receptor 4 (TLR4) extends survival in the hSOD1^G93A^ mouse model of amyotrophic lateral sclerosis

**DOI:** 10.1186/s12974-015-0310-z

**Published:** 2015-05-13

**Authors:** Jia Y Lee, John D Lee, Simon Phipps, Peter G Noakes, Trent M Woodruff

**Affiliations:** School of Biomedical Sciences, The University of Queensland, St Lucia, Brisbane, QLD 4072 Australia; Australian Infectious Diseases Research Centre, The University of Queensland, St Lucia, Brisbane, QLD 4072 Australia; Queensland Brain Institute, The University of Queensland, St Lucia, Brisbane, QLD 4072 Australia

**Keywords:** HMGB1, TLR4, Motor neuron disease, Neuro-inflammation

## Abstract

**Background:**

Amyotrophic lateral sclerosis (ALS) is a devastating late onset neurodegenerative disorder that is characterised by the progressive loss of upper and lower motor neurons. The mechanisms underlying ALS pathogenesis are unclear; however, there is emerging evidence the innate immune system, including components of the toll-like receptor (TLR) system, may drive disease progression. For example, toll-like receptor 4 (TLR4) antagonism in a spontaneous ‘wobbler mouse’ model of ALS increased motor function, associated with a decrease in microglial activation. This study therefore aimed to extend from these findings and determine the expression and function of TLR4 signalling in hSOD1^G93A^ mice, the most widely established preclinical model of ALS.

**Findings:**

TLR4 and one of its major endogenous ligands, high-mobility group box 1 (HMGB1), were increased during disease progression in hSOD1^G93A^ mice, with TLR4 and HMGB1 expressed by activated microglia and astrocytes. hSOD1^G93A^ mice lacking TLR4 showed transient improvements in hind-limb grip strength and significantly extended survival when compared to TLR4-sufficient hSOD1^G93A^ mice.

**Conclusion:**

These results suggest that enhanced glial TLR4 signalling during disease progression contributes to end-stage ALS pathology in hSOD1^G93A^ mice.

## Introduction

Amyotrophic lateral sclerosis (ALS) is an adult onset neurodegenerative disease, which is characterised by the irreversible loss of upper and lower motor neurons in the motor cortex, brainstem and spinal cord [1]. This selective loss of neurons leads to muscle denervation and atrophy, resulting in paralysis and eventual death via respiratory muscle failure [2]. The mechanisms underlying ALS pathogenesis are still unclear, but an emerging body of evidence suggests that immune and inflammatory factors could contribute to the progression of the disease [3-5]

The toll-like receptor (TLR) system is one of the major components of the innate immune system, which has been implicated in ALS pathology. Toll-like receptor 4 (TLR4) is a canonical pro-inflammatory TLR expressed by numerous immune and nonimmune cells, including cells within the central nervous system [6,7]. TLR4 activation induces the release of cytokines such as tumour necrosis factor-α and interleukins, which have been shown to be involved in ALS pathogenesis [8,9]. Several TLRs, including TLR4, are up-regulated in hSOD1^G93A^ mice suggesting an involvement in ALS disease progression [10]. Extracellularly released high-mobility group box 1 (HMGB1) is an endogenous ligand for TLR4, which is shown to translocate from the nucleus to cytoplasm in reactive astrocytes and activated microglia cells in ALS patients [11]. In addition, it was shown that TLR4 antagonism increased motor function in a spontaneous ‘wobbler’ mouse model of ALS [8]. However, the specific function of TLR4 in the most widely used preclinical hSOD1^G93A^ mouse model of ALS has yet to be reported.

In the present study, we addressed this by examining the expression of HMGB1 and TLR4 during defined stages in hSOD1^G93A^ mice. In order to identify the contribution of TLR4 signalling in hSOD1^G93A^ pathogenesis, we generated hSOD1^G93A^ mice lacking TLR4 and compared them to hSOD1^G93A^ mice in respect to survival and muscle strength. Our findings demonstrate that lack of TLR4 signalling has a protective effect on hSOD1^G93A^ pathology, significantly extending survival and transiently improving motor function.

## Methods

### Animals

Transgenic hSOD1^G93A^ mice (B6-Cg-Tg (SOD1-G93A)1Gur/J) were obtained from the Jackson laboratory (Bar Harbor Maine, USA) and were bred on C57BL/6J background to produce hSOD1^G93A^ and wild-type (WT) mice. Female hSOD1^G93A^ and WT mice at four predefined stages of ALS were used in this study as described previously [12]. Specifically, the predefined stages of disease progression are: (1) *presymptomatic* at 30 days postnatal where no motor deficits are seen, (2) *onset* at 70 days postnatal where there is initial signs of motor deficits determined by a significant reduction in hind-limb grip strength, (3) *mid-symptomatic* at 130 days postnatal where there is marked weakness in hind-limbs and tremor when suspended by the tail, and (4) *end-stage* at 150 to 175 days postnatal where there is full paralysis of lower limbs and loss of righting reflex (also defined as the survival end-point). TLR4^−/−^ female mice on C57BL/6 background, a gift originally from Dr. Shizuo Akira, were bred with male hSOD1^G93A^ to yield hSOD1^G93A^ mice lacking TLR4 (hSOD1^G93A^ × TLR4^−/−^) at F2 generation. Female hSOD1^G93A^ and hSOD1^G93A^ × TLR4^−/−^ mice were used for all phenotype studies. All experimental procedures were approved by the University of Queensland Animal Ethics Committee.

### Survival analysis, weight measurements and hind-limb grip strength test

Survival was determined by the inability of the animal to right itself within 30 s if laid on either side. This is a widely accepted and published end-point for life span studies in ALS mice [13,14]. The weight and hind-limb grip strength of hSOD1^G93A^ and hSOD1^G93A^ × TLR4^−/−^ mice were measured as described previously [12].

### Quantitative PCR

Gene expression was measured by SYBR Green real-time PCR (Applied Biosystems, Grand Island, NY, USA) according to manufacturer’s protocols. All primers used are listed in Table 1. Final measures are presented as relative levels of gene expression in hSOD1^G93A^ mice compared with expression in WT as described previously [12].

**Table 1:** **List of primers used for SYBR Green quantitative PCR**

**Gene of interest**	**Primer sequence**	**Product length**
TLR4	Forward: 5′ - ATGCATGGATCAGAAACTCAGCAA - 3′	249
	Reverse: 5′ - AAACTTCCTGGGGAAAAACTCTGG - 3′	
HMGB1	Forward: 5′ - GCTCTCACAGCCATTGCAGTACAT - 3′	129
	Reverse: 5′ - AGGATCTCCTTTGCCCATGTTTAG - 3′	
GAPDH	Forward: 5′ - AGGTCGGTGTGAACGGATTTG - 3′	123
	Reverse: 5′ - TGTAGACCATGTAGTTGAGGTC - 3′	

TLR4, toll-like receptor 4; HMGB1, high-mobility group box 1; GAPDH, glyceraldehyde 3-phosphate dehydrogenase.

### Western blotting

Lumbar spinal cord homogenates were resolved on a 10% sodium dodecyl sulphate polyacrylamide gel and electro-transferred onto nitrocellulose membranes. The membrane was incubated with anti-TLR4 (1:500; Santa Cruz Biotechnology, Dallas, Texas, USA) or anti-HMGB1 (1:1000; Abcam, Melbourne, Victoria, Australia) antibodies and were detected with enhanced chemiluminescence (GE Healthcare, Sydney, New South Wales, Australia). Densitometric analyses of immunoreactive bands were quantified as described previously [12].

### Immunohistochemistry

Fluorescence double immunolabelling was performed to localise the expression of TLR4 with specific cell-type markers for motor neurons, astrocytes and microglia as described previously [12]. The combination of antibodies used in this study is outlined in Table 2.

**Table 2:** **Summary of antibodies used for immunohistochemistry**

**Antibody**	**Manufacturer**	**Dilution**	**In combination with**
Rat anti-mouse TLR4	R & D Systems	1:500	GFAP, Iba-1 and ChAT
Rabbit anti-mouse HMGB1	Abcam	1:1,000	GFAP, CD11b and ChAT
Mouse anti-mouse GFAP	BD Biosciences	1:1,000	TLR4
Rabbit anti-mouse Iba-1	Wako	1:400	TLR4
Goat anti-mouse ChAT	Chemicon	1:100	TLR4

TLR4, toll-like receptor 4; HMGB1, high-mobility group box 1; GFAP, glial fibrillary acidic protein; ChAT, choline acetyltransferase; Iba-1, ionised calcium-binding adapter molecule-1.

### Statistical analysis

Statistical differences between hSOD1^G93A^ and hSOD1^G93A^ × TLR4^−/−^ mice were analysed using a two-tailed *t*-test at each time point and stage of disease progression and a log-rank test for Kaplan-Meier survival plots. All data are presented as mean ± SEM and differences were considered significant when *P* ≤ 0.05.

## Results

### HMGB1 and TLR4 are up-regulated during disease progression in hSOD1^G93A^ mice

**Figure 1:**
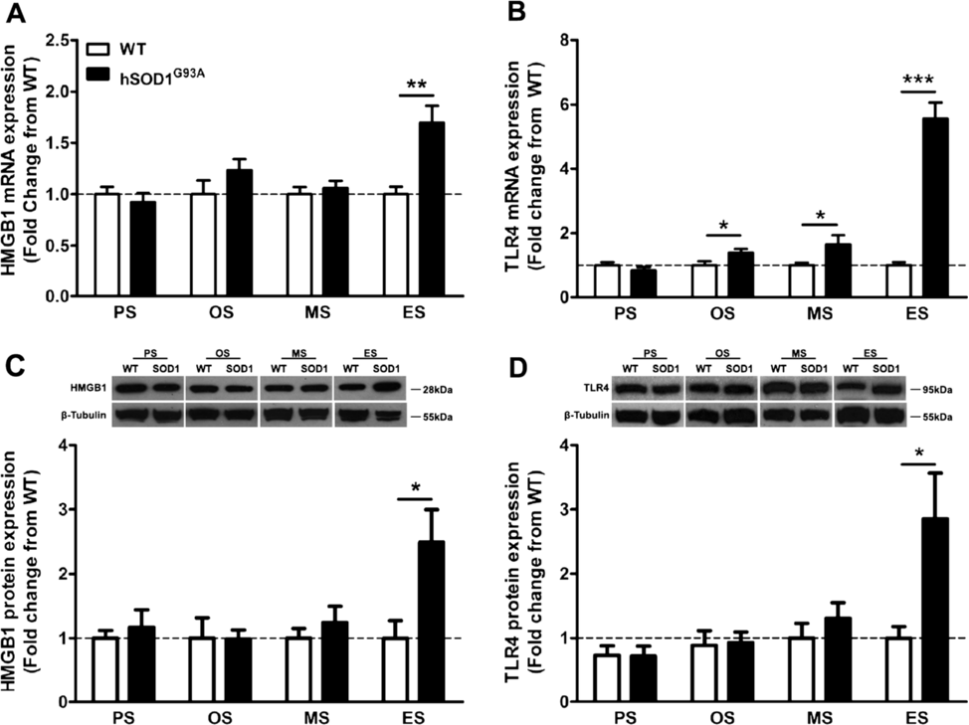
Expression of HMGB1 and TLR4 during disease progression in wild-type and hSOD1^G93A^ mice. **(A)** and **(B)** mRNA expression profile of HMGB1 and TLR4 in the lumbar spinal cord of hSOD1^G93A^ mice relative to wild-type (WT) mice at four disease stages. **(C)** Representative Western blot of HMGB1 with β-tubulin in the lumbar spinal cord of hSOD1^G93A^ (SOD1) mice relative to WT mice at four disease ages. Protein expression of HMGB1 determined by semi-quantitative densitometry in the lumbar spinal cord of hSOD1^G93A^ (SOD1) mice relative to WT mice at four different ages. **(D)** Representative Western blot of TLR4 with β-tubulin in the lumbar spinal cord of hSOD1^G93A^ (SOD1) mice relative to age-matched WT mice at different ages. Protein expression of TLR4 determined by semi-quantitative densitometry in the lumbar spinal cord of hSOD1^G93A^ (SOD1) mice relative to age-matched WT mice at four different ages. Data expressed as mean ± SEM (*n* = 9 mice/group **(A)** and **(B)**; *n* = 3~4 mice/group **(C)** and **(D)**; *P < 0.05, **P < 0.01, ***P < 0.001, Student’s *t*-test). Dashed line represents the baseline expression in WT mice at each disease stage. PS = pre-symptomatic; OS = onset; MS = mid-symptomatic; ES = end-stage; HMGB1 = high-mobility group box 1; mRNA = messenger RNA; TLR4 = toll-like receptor 4.

We initially examined the mRNA expression of TLR4 and HMGB1 in the lumbar spinal cord during key disease stages in hSOD1^G93A^ mice. HMGB1 mRNA levels were increased by 1.7 fold at the end-stage of disease, compared with WT mice (*n* = 9, **P < 0.01; Figure 1A). TLR4 mRNA in hSOD1^G93A^ mice progressively increased by 1.4 fold, 1.6 fold and 5.6 fold at onset, mid-symptomatic and end-stage, respectively, when compared to WT mice (*n* = 9, *P < 0.05 and ***P < 0.001; Figure 1B). At the protein level, HMGB1 protein expression increased at the end-stage of disease (2.5 fold increase; *n* = 4, *P < 0.05; Figure 1C). TLR4 protein expression was also increased by 2.9 fold at the end-stage of disease when compared with WT mice (*n* = 4, *P < 0.05; Figure 1D).

### HMGB1 and TLR4 are expressed by ALS-relevant cell types in hSOD1^G93A^ mice

**Figure 2:**
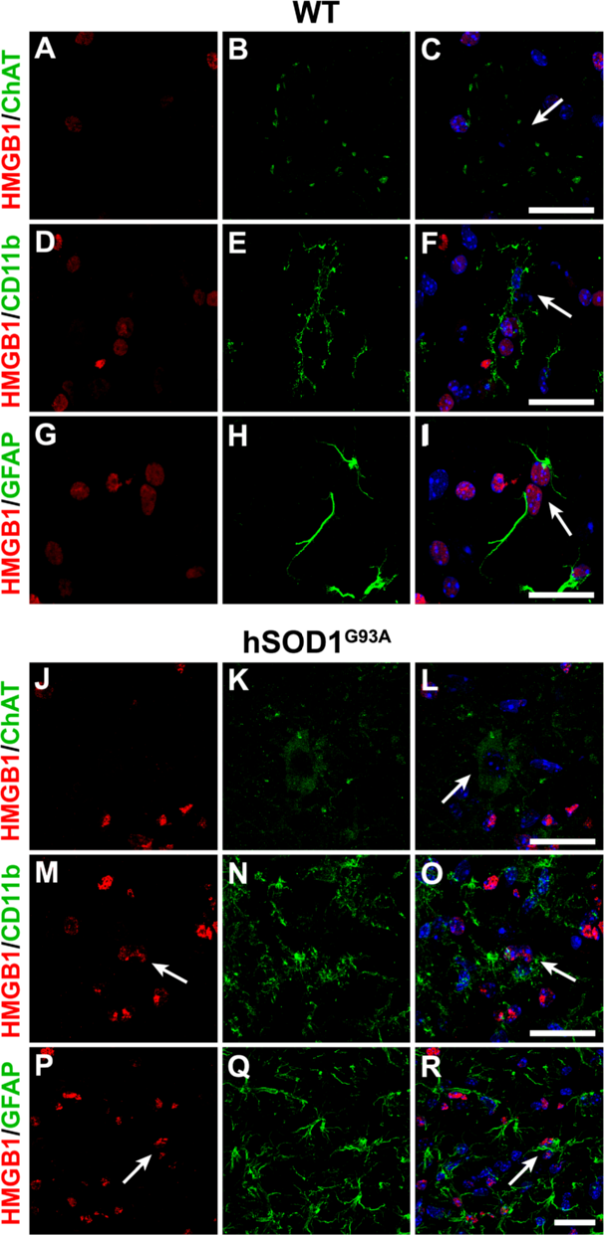
Localization of HMGB1 in wild-type and hSOD1^G93A^ mice at end-stage of disease. **(A-R)** Double immunolabelling of HMGB1 (red) with cellular markers (green) for motor neurons (ChAT; **(A-C)** wild-type (WT) mice, (J-L) hSOD1^G93A^ mice), microglia (CD11b; **(D-F)** WT mice, (M-O) hSOD1^G93A^ mice), and astrocytes (GFAP; (G-I) WT mice, (P-R) for hSOD1^G93A^ mice) in the ventral lumbar spinal cord of WT and hSOD1^G93A^ mice at end-stage of disease. HMGB1 displayed diffuse nuclear staining and was mainly co-localised with CD11b-labelled microglia and GFAP-positive astrocytes in WT mice (F, I, white arrows), with no co-localisation with ChAT-positive motor neurons (C). In hSOD1^G93A^ mice, HMGB1 immunolabelling increased in intensity and appeared more punctate and was evident on GFAP-positive astrocytes and CD11b-labelled microglia (white arrows in M, O, P, R). Similar to WT mice, immunolabelling of HMGB1 was absent from ChAT-positive motor neurons in hSOD1^G93A^ mice (L, white arrow). Scale bars for all panels = 10 μm. HMGB1 = high-mobility group box 1; ChAT = choline acetyltransferase; CD11b = cluster of differentiation molecule 11B; GFAP = glial fibrillary acidic protein.

**Figure 3:**
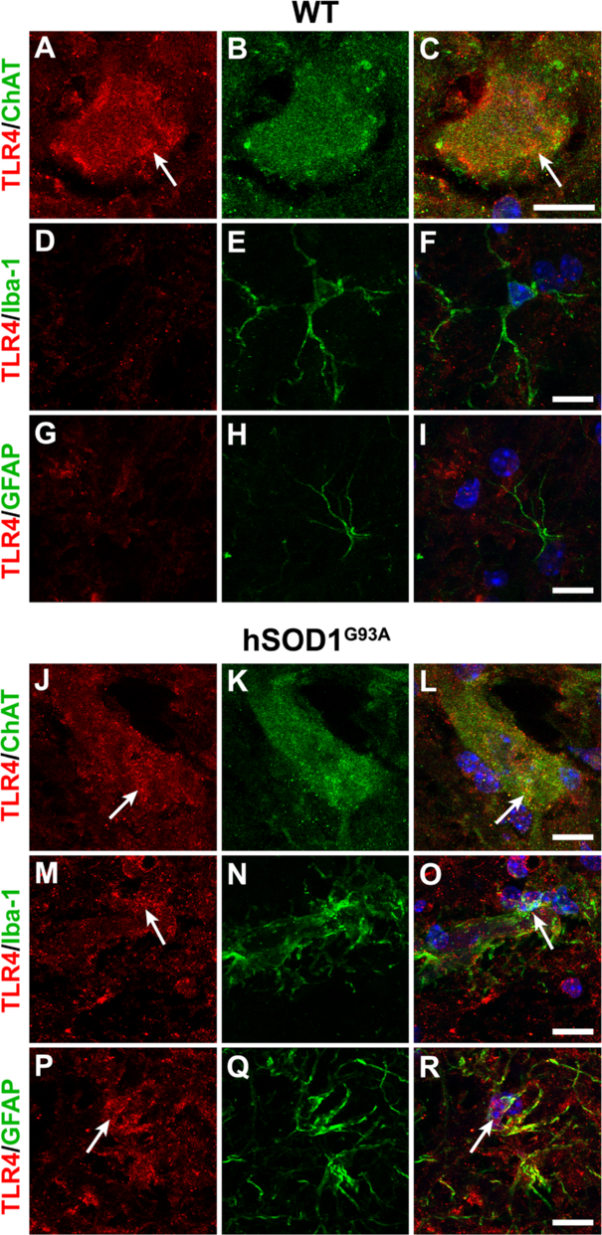
Localization of TLR4 in wild-type and hSOD1^G93A^ mice at end-stage of disease. **(A-R)** Double immunolabelling of TLR4 (red) with cellular markers (green) for motor neurons (ChAT; (A-C) wild-type (WT) mice, (J-L) hSOD1^G93A^ mice), microglia (Iba-1; (D-F) WT mice, (M-O) hSOD1^G93A^ mice), and astrocytes (GFAP; (G-I) WT mice, (P-R) for hSOD1^G93A^ mice) in the ventral lumbar spinal cord of WT and hSOD1^G93A^ mice at end-stage of disease. TLR4 was mainly co-localised with ChAT-positive motor neurons in the WT mice (A, C, white arrow) with minimal co-localisation with Iba-1-labelled microglia and GFAP-positive astrocytes (F, I). In hSOD1^G93A^ mice, TLR4 immunolabelling was evident on ChAT positive motor neurons and GFAP-positive astrocytes with some localisation with Iba-1-labelled microglia (white arrows in J, L, M, O, P, R). Scale bars for all panels = 10 μm. TLR4 = toll-like receptor 4; ChAT = choline acetyltransferase; Iba-1 = ionised calcium-binding adapter molecule-1; GFAP = glial fibrillary acidic protein.

Next, we immuno-stained the lumbar spinal cords from hSOD1^G93A^ and WT mice for HMGB1 and TLR4 with specific cellular markers for motor neurons (anti-choline acetyltransferase(ChAT)), microglia (anti-CD11b/anti-ionised calcium-binding adapter molecule-1(Iba-1)) and astrocytes (anti-glial fibrillary acidic protein (GFAP)). HMGB1 was expressed by CD11b-positive microglia and GFAP-positive astrocytes in WT (white arrows in Figure 2F, I) and hSOD1^G93A^ mice (white arrows in Figure 2M, O, P, R). However, HMGB1 did not significantly co-localise with motor neurons in both WT and hSOD1^G93A^ mice (white arrows in Figure 2C,L). By contrast, TLR4 was primarily expressed on motor neurons in WT mice (white arrows in Figure 3A,C) and did not co-localise with surrounding Iba-1-positive microglia (Figure 3D,E,F) or GFAP-positive astrocytes (Figure 3G,H,I). In hSOD1^G93A^ mice, TLR4 was still expressed by the few remaining motor neurons at the end-stage of disease (white arrow in Figure 3J,L). However, as compared to WT mice, in hSOD1^G93A^ mice, TLR4 was strongly expressed on GFAP-positive astrocytes (white arrows in Figure 3P,R) with some localisation with Iba-1-labelled microglia (white arrows in Figure 3M,O).

### hSOD1^G93A^ mice lacking TLR4 transiently improves hind-limb grip strength and extends survival when compared to hSOD1^G93A^ mice

**Figure 4:**
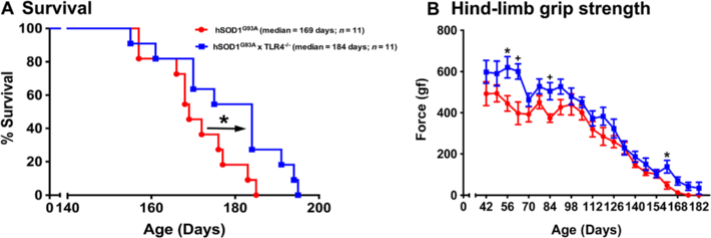
hSOD1^G93A^ mice lacking TLR4 (hSOD1^G93A^ × TLR4^−/−^) have extended survival and improvements in hind-limb grip strength when compared to hSOD1^G93A^ mice. **(A)** shows a Kaplan-Meier plot of ages (in days) in which hSOD1^G93A^ mice with normal (TLR4^+/+^, red line) or fully deleted (TLR4^−/−^, blue line) TLR4 reached the end-stage of disease (complete hind-limb paralysis and an inability to right itself once placed on its back). hSOD1^G93A^ × TLR4^−/−^ shows a significant extension in survival time relative to hSOD1^G93A^ mice (*n* = 11, *P* < 0.05, log-rank test). **(B)** shows hind-limb grip strength in these two groups. A small overall improvement in grip strength was seen in hSOD1^G93A^ × TLR4^−/−^ mice versus hSOD1^G93A^ mice, with significant differences observed at 56, 63, 84 and 161 days of age (*n* = 8–9, *P < 0.05, +*P* < 0.01, Student’s *t*-test). Data are expressed as mean ± SEM.

Given our findings demonstrating increased expression of TLR4 and one of its endogenous ligands HMGB1, in hSOD1^G93A^ mice, we next assessed whether enhanced TLR4 signalling contributes to disease pathogenesis by generating hSOD1^G93A^ mice lacking TLR4 (hSOD1^G93A^ × TLR4^−/−^). hSOD1^G93A^ × TLR4^−/−^ mice showed extended survival when compared to hSOD1^G93A^ mice (median end-stage of disease, hSOD1^G93A^ = 169 days and hSOD1^G93A^ × TLR4^−/−^ = 184 days, *n* = 11, *P < 0.05; Figure 4A). Concomitant with enhanced survival, there were significant improvements in hind-limb grip strength of hSOD1^G93A^ × TLR4^−/−^ mice when compared to hSOD1^G93A^ mice at 56, 63, 84 and 161 days of age (*n* = 8 to 9, *P < 0.05, +*P* < 0.01; Figure 4B). No differences in body weights were seen between the groups at any age (data not shown).

## Discussion

Although the exact mechanisms that underlie the pathogenesis of ALS remain unclear, there is credible evidence that a co-ordinated action of innate and adaptive immune factors is involved in the progression of ALS. This includes evidence for major innate immune systems such as the complement cascade [12,15] and the toll-like receptor system [16]. For example, the pathogenic effect of TLR4 has been observed in the wobbler mouse model of ALS, where TLR4 antagonism improved disease outcomes [8]. The present study adds additional support to a pathogenic role for TLR4 in ALS.

We identified TLR4 up-regulation in the lumbar spinal cord of hSOD1^G93A^ mice, where it was localised predominantly to astrocytes and some microglia. Importantly, HMGB1, an endogenous cell secreted ligand for TLR4, was similarly up-regulated and expressed by astrocytes and microglia. It is plausible that degenerating motor neurons and the associated neuroinflammatory process triggers HMGB1 release from activated astrocytes and microglia in hSOD1^G93A^ mice. This, in turn, could trigger further neuronal death via binding to glia-expressed TLR4 to release additional neurotoxic factors, although this hypothesis is yet to be tested in this disease model. In support of this, activation of TLR4 on microglia and astrocytes can trigger different signalling pathways that lead to the production of pro-inflammatory mediators including cytokines, nitric oxide and reactive oxygen species [17]. Taken together, this suggests that TLR4 signalling could be an additional player in the pro-inflammatory reactions that exacerbate disease progression in ALS, as shown previously in ischemic stroke [18] and Alzheimer’s disease [19].

To identify the role TLR4 plays in ALS disease progression, we generated hSOD1^G93A^ mice lacking TLR4 (hSOD1^G93A^ × TLR4^−/−^). We observed that deletion of the TLR4 gene in hSOD1^G93A^ mice significantly extended survival when compared to hSOD1^G93A^ mice expressing TLR4. This was accompanied by significant improvements in hind-limb grip strength at select time points. This supports the hypothesis that enhanced TLR4 signalling contributes to ALS progression and that this effect likely occurs through increased activation of microglia and astrocytes [8,20]. It should be noted however, that the extent of survival extension, and motor functional improvements in hSOD1^G93A^ mice were moderate, indicating that TLR4 is but one of many contributors to hSOD1^G93A^ ALS disease pathogenesis.

In summary, the present study has demonstrated that deletion of TLR4 significantly extends survival and transiently improves hind-limb grip strength in an ALS disease model, suggesting that TLR4 signalling in these animals may contribute to motor neuron death and ultimately disease progression of ALS. Hence, this study suggests that reducing downstream consequences of TLR4 activation through specific inhibitors should be further explored as one potential therapeutic strategy to slow disease progression in ALS.

## Abbreviations

ALS: amyotrophic lateral sclerosis
HMGB1: high-mobility group box 1
TLR: toll-like receptor
WT: wild-type
